# Death of M. Berger

**Published:** 1856-03

**Authors:** 


*Died*—At Brussels, M. Berger, Dean of the Accoucheurs of that city, who is said to have delivered at least 20,000 women.

At Paris, M. Louis Gondret, aged 79, known by his advocacy of the use of ammoniacal preparations in amaurosis, etc.

At Vienna, after a short illness, in the 64th year of his age, Dr. A. de Rosas, Professor of Ophthalmology and Director of the Ophthalmic Clinic of the University. He spent thirty-six years in teaching his speciality.—*Counselor.*

